# The Change of Metallothionein and Oxidative Response in Gills of the *Oreochromis niloticus* after Exposure to Copper

**DOI:** 10.3390/ani9060353

**Published:** 2019-06-14

**Authors:** Faridlotul Ma’rifah, Miftahul Rohmah Saputri, Agoes Soegianto, Bambang Irawan, Trisnadi Widyaleksono Catur Putranto

**Affiliations:** Department of Biology, Faculty of Sciences and Technology, Universitas Airlangga, Surabaya 60115, Indonesia; rifah_farida@yahoo.co.id (F.M.); miftahulrohmahsaputri@gmail.com (M.R.S.); bamir1955@yahoo.co.id (B.I.); widyaleksono.cp@gmail.com (T.W.C.P.)

**Keywords:** cichlid fish, Cu, metallothionein, oxidative stress, free radicals

## Abstract

**Simple Summary:**

Copper is an essential element for the aquatic organisms for a number of biological processes. However, it may be toxic at high concentrations. The present study revealed that the levels of Cu in gills of all Cu-exposed tilapia significantly increased during the first few days, and then gradually decreased, matching the control at D4-D5. The concentration of metallothionein (MT) and the activities of superoxide dismutase (SOD) and catalase (CAT) in the gills of Cu-exposed fish were in line with the accumulated Cu. The increase of MT, SOD, and CAT during the first few days might be the adaptive response of the animal to Cu toxicity. MT binds the elevated Cu, while SOD and CAT scavenge the increased free radicals due to the increasing level of Cu. Cu does not affect the malondialdehyde (MDA) concentration in gills of fish, which suggests the SOD, CAT and MT as antioxidant defense systems were able to completely scavenge the increased free radicals.

**Abstract:**

In the present study, we investigated the effects of waterborne copper (Cu) on the levels of metallothionein (MT) and malondialdehyde (MDA), as well as activities of superoxide dismutase (SOD) and catalase (CAT) in gills of cichlid fish *Oreochromis niloticus*. The Cu concentrations in gills were measured using an atomic absorption spectrometer. The sandwich-ELISA was used to measure MT, SOD, CAT, and MDA. The Cu concentrations in gills of fish that were exposed to 1, 5, and 10 mg Cu/L were significantly increased at day 1 (D1), then gradually decreased starting from D2, and reaches the similar value with the controls at D5. A similar tendency has been observed in the MT levels in the gills. All of the Cu-exposed fish showed the highest level of MT on D1, and then decreased at D3 and a plateau at D4 and D5. The levels of SOD and CAT in gills in all Cu-exposed fish showed a similar pattern: increased significantly at D1, then gradually decreased starting from D2, and increased again at D4 and D5. The levels of MDA in gills of all Cu-exposed fish showed no significant difference. The indifference levels of MDA in gills of all Cu-exposed fish suggested the antioxidant defense systems (SOD and CAT) combined with the induction of MT were able to completely scavenge the increased ROS.

## 1. Introduction

Copper is commonly found in aquatic systems as a result of both natural (such as geological deposits, volcanic activity, weathering and erosion of rocks and soils), and anthropogenic sources (such as mining activities, agriculture, metal and electrical manufacturing, and pesticide use) [1]. In most natural waters, copper is usually present in sublethal concentrations; however, in polluted waters, it can reach 0.85–7.75 mg/L [2,3,4,5], and have, in some cases, been reported as higher as 200 mg/L in and near mining areas [1,6,7].

Copper is an essential element for aquatic organisms, including fish [8,9]. It can be utilized by a number of processes, such as cellular respiration, iron oxidation, pigment formation, connective-tissue biogenesis, peptide amidation, neurotransmitter biosynthesis, and free-radical defense and cellular metabolism [10,11]. However, Cu is also a very toxic element when its cellular level is elevated [11,12]. Therefore, maintaining the homeostasis of body Cu levels needs to be done in order to guard against Cu deficiency and toxicity [13,14]. The maintenance of body Cu balance involves the strict regulation of uptake, distribution, metabolism, detoxification, and excretion [13,15].

Freshwater fish uptake waterborne Cu mainly through the gills, followed by the skin and intestine [16]. From toxicological studies it has been demonstrated that elevated copper concentration in water can lead to increased copper levels in the gills [17,18], and it alters the function of the gills by causing severe ion regulation, gas exchange, and excretion of metabolic waste products [19,20,21,22,23,24]. 

The accumulation of metals in organs does not always cause harmful effects so long as the organisms are able to protect themselves from metal toxicity by increasing excretion, sequestering among organs and by intercellularly binding metals with metallothionein (MT) [25,26]. Studies suggest that antioxidants may also play an important role in reducing hazardous effects of metals [27,28,29]. Antioxidant enzymes superoxide dismutase (SOD), glutathione peroxidase (GPx), and catalase (CAT) are considered to be vital defenses against reactive oxygen species (ROS) toxicity due to the change in metal levels [25,28,29,30,31]. However, when the production of ROS surpasses the capacity of cells to remove them, excessive ROS can provoke oxidative stress, which induces lipid peroxidation (LPO). Malondialdehyde (MDA) is one of the final products of LPO and it is responsible for cell membrane damage [31,32,33]. 

In the present study, we investigated the concentrations of MT and MDA, as well as the activities of SOD and CAT in the gills of fish *Oreochromis niloticus* after exposure to waterborne copper.

## 2. Materials and Methods

### 2.1. Fish Acclimation

The fish *O. niloticus*, which were approximately 10.7 ± 0.4 cm length and 15.2 ± 0.6 g, weight were obtained from a commercial farm in Pasuruan, East Java Province, Indonesia. The fish were transferred to the laboratory of Department of Biology, Universitas Airlangga, Indonesia, and kept for two weeks for adaptation in a large fiberglass tank (250 L) that was supplied with a continuous flow of dechlorinate freshwater (FW) through gravel, sand, and sponge filter. Illumination was maintained at 12 h light, with a 12 h dark cycle using fluorescent tubes as a light sources. During the acclimation fish were fed twice a day to satiation with Takari commercial pellets (30% protein, 3% fat, and 4% fiber). The experiments in the present study were conducted in accordance with the principles and procedures that were approved by the Institutional Animal Care of Universitas Airlangga.

### 2.2. Determination 96-h LC_50_ of Copper

Before used as experimental animals, the fish were starved 48 h. A static bioassay was performed to determine the median lethal concentration (96h L C_50_) of Cu to *O. niloticus*. Fish were exposed to nominal copper concentrations: 0 (control), 2.5, 5, 10, 20, 40, and 80 mg/L in 63 L plastic tanks (each tank contains 50 L of testing media). We used a total of 140 *O. niloticus* with 10 fish per tank and two tanks per group. A stock solution of Cu (1000 mg/L) was prepared by dissolving 3.845 g CuSO_4_.5H_2_O (Merck, Germany) in 1000 mL deionized water. During the toxicity test, media were continuously aerated, but were not renewed. The fish were not fed during the toxicity test. Regular observations were made, and fish mortality was recorded daily. The 96 h LC_50_ and 95% confidence intervals were calculated while using the trimmed Spearman–Karber method [34]. The 96 h LC_50_ and 95% confidence intervals of Cu to *O. niloticus* were 26.02 (20.89–32.41) mg/L. The Cu concentrations that were used in this study were 1, 5, and 10 mg/L. According to the ecotoxicological point of view, these concentrations can potentially be found by fish in their natural environment [1].

### 2.3. Treatment with Sublethal Cu

After acclimation, 160 individuals were randomly selected from the holding tank and distributed among 32 tanks, with five fish per each tank. Each tank contains 50 L of selected testing media: 1, 5, and 10 mg Cu/L, and the control (without Cu). There were two tanks per concentration and time. The experiment was conducted in a static system with 80% of test solutions being renewed every 48 h. The Cu treatment was conducted for 120 h, gills samplings were done at the intervals of 24 h (D1 group), 48 h (D2 group), 72 h (D3 group), 96 h (D4 group), and 120 h (D5 group) using five fish that were randomly collected from two tanks of Cu treatment, while gills sampling from the control group was conducted after 24 h (Table 1). The gills of fish in the control group were not sampled daily, since the concentration will not change. Before being euthanized, fish were anesthetized with 200 mg/L clove solution [35], then the gills were sampled for MT, SOD, CAT, and MDA measurement. The detail of gills preparation was described in Section 2.5 and Section 2.6.

### 2.4. Water Quality

The temperature was measured using mercury-in-glass thermometer (°C), pH while using a pH meter (Hanna Model HI 98150, Beijing, China), and the dissolved oxygen (DO) using the DO meter (Lutron DO 5510, Taiwan). The values of temperature, pH, and dissolved oxygen during the experiment were 27–29 °C, 7.65–8.10, and 7.0–7.5 mg/L, respectively.

### 2.5. Determination of Cu 

After the fish were sacrificed, the gills were dissected and stored at −20 °C until a Cu determination, as described by Ruaeny et al. [36]. Prior to the analysis, the gills were thawed at room temperature and then kept in the oven at 65 °C for 48 h to a constant weight. The dried sample was then ground into a fine powder using agate mortar and pestle. Approximately 2 g of the homogenized tissue sample was thoroughly homogenized and digested while using 5 mL concentrated HNO_3_ at 100 °C for 3 h in the microwave digester (Mars 6, CEM Corporation, North Carolina, USA). After cooling, the sample was diluted to 50 mL with deionized water. An aliquot was taken for Cu detection while using an atomic absorption spectrometer (ZEEnit 700, Analytik Jena AG, Jena, Germany). The Cu concentrations were expressed as mg/kg dry weight (dw). Analytical blanks were run in the same way as the samples, and the concentrations were determined using standard solutions that were prepared in the same acid matrix. The accuracy of the Cu determination was verified using dogfish muscle reference material (DORM-2) provided by the National Research Council of Canada (Ottawa, Canada), with Cu recovery of 109% and a detection limit of 0.002 mg/kg dw.

### 2.6. Determination of MT, SOD, CAT and MDA 

The sandwich-ELISA was used to measure MT, SOD, CAT, and MDA. All the micro-titer plates that were provided in the kits were pre-coated with an antibody specific to MT, SOD, CAT, and MDA, respectively. The measurement protocol followed the instructions from Bioassay Technology Laboratory (Shanghai, China), as described below.

On the last day of the experiment, the fish were sacrificed, the gills were carefully excised, weighed, and minced into small pieces, rinsed thoroughly in ice-cold phosphate-buffered saline (PBS, 0.01 M, pH = 7.4) to remove excess blood, and stored at −20 °C until analysis (≤5 days). One gram of tissue pieces were weighed and homogenized in PBS with a motorized mini handheld homogenizer on ice. The homogenates were then centrifuged at 3000 RPM for 20 min to obtain the supernatant. 

To measure the levels of MT and MDA, and the activities of SOD and CAT, all of the samples experienced the following treatments: 50 μL of standard and 40 µL sample was added to each well, then immediately 10 µL of a biotin-conjugated anti-Fish (MT, MDA, SOD, and CAT, respectively) antibody was added to each well, and then 50 µL of streptavidin horseradish was added to the sample and standard well and mixed well. The plate was then covered with a sealer that was provided by kit manufacture and incubated for 60 min at 37 °C.

After conducting the above procedure, the plates were aspired, washed five times with wash buffer, and then soaked with wash buffer (approximately 350 µL/well) for one minute. Any remaining wash buffer was removed by aspirating or decanting. Further, 50 µL of substrate solution A and 50 µL of substrate solution B were added to each well. Subsequently, the wells were covered with new sealers and incubated for 10 min at 37 °C in the dark. To terminate the enzyme reaction, 50 μL of stop solution was added to each well, and the blue color would immediately change into yellow. The optical density of each well was determined using an automatic microplate reader (Bio-Rad, model iMark, Japan) at 450 nm within 10 min after adding the stop solution. The concentrations of MT and MDA, and the activities of SOD and CAT were determined while using the appropriate standard curves and data were expressed as ng/mL for MT and SOD, mU/mL for CAT, and nmol/mL for MDA. Afterwards, all data should be normalized to the weight of the gills.

### 2.7. Statistical Analysis

The data were tested for distribution using the Kolmogorov–Smirnov test. Subsequently, the data were subject to two-way ANOVA to evaluate the effects of waterborne Cu exposure, time, and Cu-time interaction on the level of Cu, MT, and MDA, as well as the activities of SOD and CAT in gills, respectively. Tukey’s test was employed for multiple means comparisons at a significance level of 0.05 when significant differences were detected (*p* < 0.05).

## 3. Results

**Figure 1:**
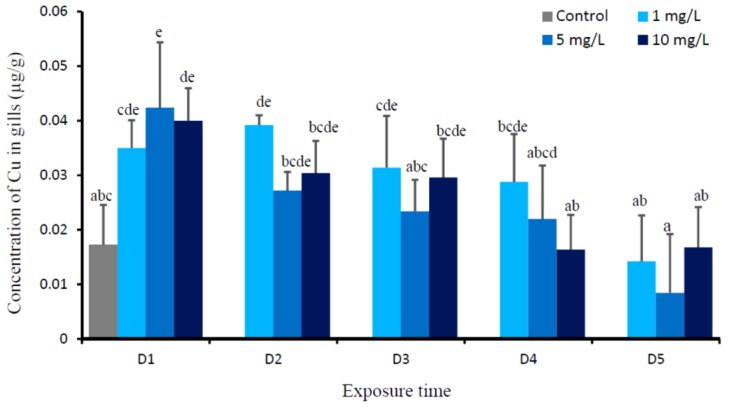
Mean concentration of Cu (µg/g) in gills of *O. niloticus* of the control group and after exposed to 1, 5, and 10 mg Cu/L. Different lowercase letter indicates significance difference according to Tukey multiple comparison test (*P* < 0.05).

No fish mortality was noted during the experiments. Two-way ANOVA revealed that there were significant Cu (*p* = 0.000), time (*p* = 0.000), and Cu-time interaction effects (*p* = 0.025) on the levels of Cu in gills of *O. niloticus*. Figure 1 presents the daily changes of Cu levels in the gills of fish after being exposed to 1, 5, and 10 mg Cu/L. The changes of Cu levels in the gills of fish that were exposed to all copper levels had almost similar pattern. When compared to the control group, the Cu concentrations in gills of all Cu-exposed fish were significantly increased at D1, then gradually decreased starting from D2 to D4, and its concentration reaches the same value with the control at D5. The levels of Cu in gills in fish exposed to 1, 5, and 10 mg Cu/L were not significantly different at the D1, D2, D3, D4, and D5 groups, respectively.

**Figure 2:**
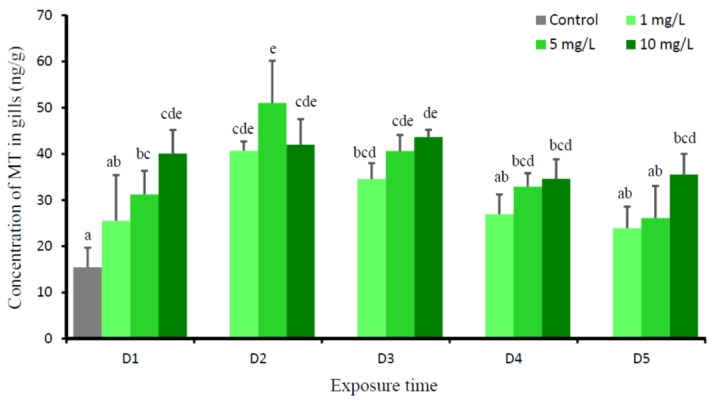
Mean concentration of metallothionein (MT) (ng/g) in gills of *O. niloticus* of the control group and after exposed to 1, 5, and 10 mg Cu/L. Different lowercase letter indicates significance difference according to Tukey multiple comparison test (*P* < 0.05).

This experiment showed the significant effects of Cu (*P* = 0.000), time (*P* = 0.000), and Cu-time interaction (*P* = 0.014) on the levels of MT in the gills of tilapia. The changes of MT levels in the gills of the fish exposed to all copper levels had relatively the same pattern. The levels of MT in gills of all Cu-exposed fish gradually increased, starting from D1, reached the highest level at D2, then decreased at D3, and reached an apparent plateau at D4 and D5. At D5, only fish that were exposed to 1 mg Cu/L were not significantly different with the control group (Figure 2).

**Figure 3:**
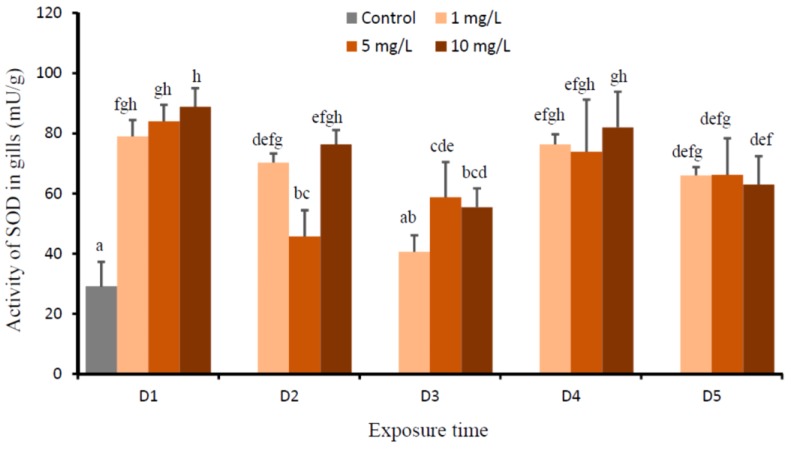
Activity of superoxide dismutase (SOD) (mU/g) in gills of *O. niloticus* of the control group and after exposed to 1, 5, and 10 mg Cu/L. Different lowercase letter indicates significance difference according to Tukey multiple comparison test (*P* < 0.05).

This experiment showed that the effects of Cu (*P* = 0.000), time (*P* = 0.000), and Cu-time interaction (*P* = 0.000) on the activities of SOD in the gills of *O. niloticus* were significant. The activities of SOD of fish that were exposed to all Cu concentrations significantly increased at D1 when compared to the control, then gradually decreased from D2 to D3, and increased again at D4 and D5 (Figure 3). The activities of SOD of all treatments on D5 were not significantly different and higher than those of the controls. The lowest SODs were demonstrated in fish that were exposed to 1 mg Cu/L at D3 and exposed to 5 mg Cu/L at D2, respectively.

**Figure 4:**
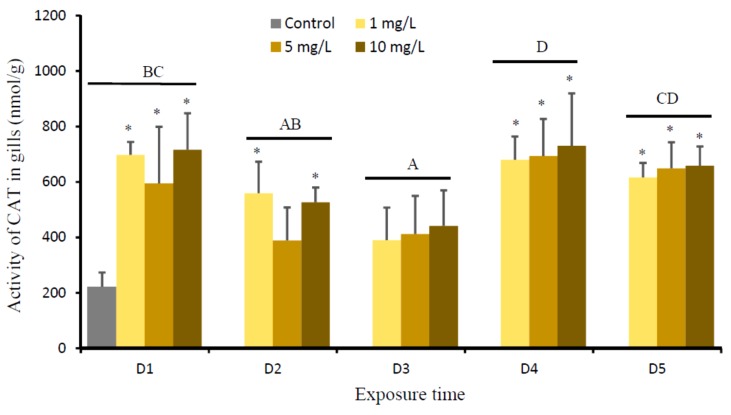
Activity of catalase (CAT) (nmol/g) in gills of *O. niloticus* of the control group and after exposed to 1, 5, and 10 mg Cu/L. Different capital letter indicates significance difference of time effect on CAT according to Tukey multiple comparison test (*P* < 0.05). Asterisk indicates significance difference of Cu effect on CAT for each time group as compared to the control (*P* < 0.05) according to Tukey multiple comparison test.

This study demonstrated that there was a significant Cu effect (*P* = 0.000) and time effect (*P* = 0.000) on the activities of CAT in gills of *O. niloticus*. However, the effects of Cu-time interaction on the activities of CAT in gills (*P* = 0.613) were not significant. When compared to the control, the activities of CAT of gills significantly increased at D1 in fish exposed to all Cu concentrations. Subsequently, the values gradually decreased from D2 to D3, and increased again at D4 and D5. The activities of CAT of all Cu-exposed fish on D5 were higher than the control group (Figure 4).

**Figure 5:**
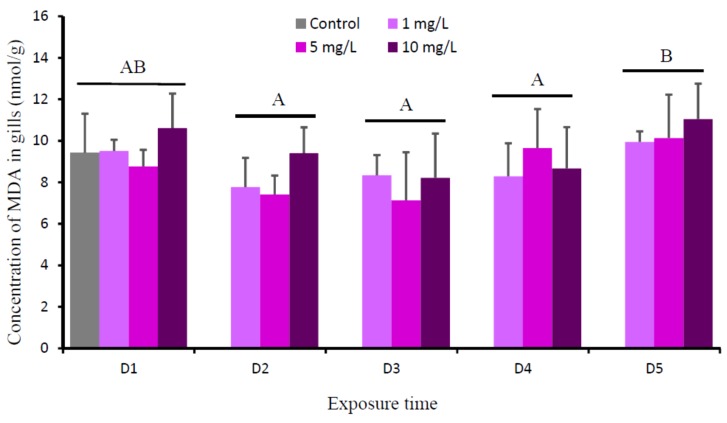
Mean concentration of MDA (nmol/g) in gills of *O. niloticus* of the control group and after exposed to 1, 5, and 10 mg Cu/L. Different capital letter indicates significance difference between the experimental day (time) for each treatment group on MDA (*P* < 0.05) according to Tukey multiple comparison test.

According to the two-way ANOVA, there were significant time effects (*P* = 0.000) on the level of MDA in the gills of tilapia. Meanwhile, the effects of Cu (*P* = 0.104) and Cu-time interaction (*P* = 0.468) on the levels of MDA in gills were not significant. The levels of MDA in gills of fish exposed to all Cu concentrations for all times showed no significant difference (Figure 5). Their levels were relatively similar to the control.

## 4. Discussion

The concentrations of Cu in the gills of fish exposed to three Cu concentrations significantly increased on the first day (D1), then gradually decreased, and reached the same value with the control on D5. The highest value of Cu in gills on the first day (D1) after exposure to all Cu concentrations could be the initial shock phase of animal in response the waterborne Cu. This phase usually corresponds to a period of physical damage, primarily occurs in the gills, and results in the disturbances of internal physiological homeostasis. This damage phase is usually short-timed [37]. In our study, depending on the level of waterborne Cu, the recovery phase starts on D2, and it takes place between two and four days; however, at D5, the Cu level in gills of all Cu-exposed fish reached the control value. A similar pattern was observed in the gills of juvenile of rainbow trout (*Oncorhynchus mykiss*), Cu uptake increased during the first 1–2 h of radiolabelled copper incubation, after 2 h, branchial ^64^Cu level considerably decreased [17]. Recovery usually begins in conjunction with the increase in biosynthetic process (such as elevated protein synthesis, mitosis), which support improving the tissue damage and to correct the physiological disruptions [37,38]. Inherent within the recovery phase is the massive production of metal binding proteins, such as MT [39,40], to impede the harmful effect of metal [38]. Hogstrand and Haux [41] prompted that, if metal ions enter the cell, the synthesis of MT will increase, and homeostasis will be restored by aggregation of metal into MT. MT has been proposed to reduce Cu toxicity in fish by sequestering Cu away from metabolically important enzyme systems [19]. The present study showed that the induction of MT in the gills was proportional with the accumulated Cu; however, its increase was one day slower than the increase of Cu in the gills. Starting from D3, the level of MT gradually declined, and showed the plateau at D4 and D5; however, at D5, its level was still higher than the control. The higher level of MT in gills of D5 than the control, which suggests that it is in a temporary state of MT imbalance, and it might indicate that MT regulation is not yet down-regulated. The present study revealed that Cu did not induce serious gill damage for at least five days of Cu exposure. Apparently MTs are involved in regulating the Cu as an essential metal, and the MT binding capacity to Cu has not been exceeded [42,43]. MT can also act as non-toxic metal reserves for metalloenzyme synthesis, protect against ionizing radiation, and play an important role as an antioxidant [44,45].

The accumulation of metal leads to enhanced ROS level, leading to the damage of cellular constituent [31,46]. In organism, ROS are continuously produced and eliminated to maintain a balance of ROS concentration. ROS are generally under the tight control of antioxidant defense system [33]. The levels of SOD and CAT as the vital first-line defenses against ROS toxicity in gills of fish that were exposed to all Cu concentrations significantly increased on D1, then gradually decreased on D2 and/or D3, and increased again on D4 and D5. The levels of SOD and CAT on D5 were still higher than the control. Our results suggest that Cu exposure might significantly increase free radicals in the first few days and lead to the production of ROS, causing oxidative stress in fish. It is likely that the increase of SOD and CAT was an adaptive response of the animal to Cu toxicity and it served to neutralize the impact of increased ROS generation [31,33,46,47]. Carvalho et al. [48] revealed that SOD and CAT had prominent roles in the scavenging of free radicals to protect the organisms from oxidative stress. SOD is an early produced antioxidant to protect the cells against superoxide anions (O_2_^•−^) by decomposing these superoxide radicals to hydrogen peroxide (H_2_O_2_) and O_2_ [48,49]. CAT decomposed H_2_O_2_ to non-toxic H_2_O and O_2_ [30,48,50]. While, the decrease of SOD and CAT on D3 may be attributed to the inactivation of these enzymes and the inhibition of de novo enzyme protein synthesis after the continuous and high production of these enzymes in the first few days in response Cu stress [51], or some other antioxidants, such as glutathione peroxidase, glutathione, and glutathione-related enzymes may be active to scavenge ROS [30,33,49,52]. Furthermore, higher levels of SOD and CAT on D5 suggested that these enzymes were not yet down-regulated, since the levels of Cu in gills on D5 were not significantly different with D0 (the control).

When the production of ROS in cell exceeds the antioxidant generation, excessive ROS can destroy biomolecules through free radical attaches to polyunsaturated fatty acid side chains in cell membranes and it leads to LPO [32]. LPO is a highly destructive process that increases the rigidity and decreases the fluidity of cellular membranes [53]. It has been reported that the levels of MDA increased as a result of oxidation of lipoprotein and lipids in cell membranes during oxidative stress [47,54]. The current results showed that the level of MDA in the gills of all Cu-exposed fish were not significantly different with the control. Regarding to the present MDA values, we suggest that, although Cu exposure resulted in the generation of ROS and antioxidant defense systems (SOD and CAT); however, these defenses, combined with the increase in the MT response, were able to completely scavenge the increased ROS, which prevent LPO.

## 5. Conclusions

The present study revealed that the levels of Cu in gills of all Cu-exposed tilapia (1, 5, and 10 mg Cu/L) significantly increased during the first few days, then gradually decreased, returning to control at D4-D5. The levels of MT, SOD, and CAT in the gills of Cu-exposed fish were parallel with the accumulated Cu. The increase of MT, SOD, and CAT during the first few days might be the adaptive response of the animal to Cu toxicity. MT binds the elevated Cu, while SOD and CAT scavenge the increased free radicals due to the increasing level of Cu. Waterborne copper, even at 10 mg/L, does not increase the MDA concentration in the gills of tilapia, suggesting the antioxidant defense systems (SOD and CAT) combined with the induction of MT were able to completely scavenge the increased ROS, which prevent LPO.

## Figures and Tables

**Table 1:** The experimental design and number of fish analyzed from each experiment group.

Copper Concentration (mg/L)	Number of Tanks Used	Treatment Day (Fish Sampled/Total Fish)				
		D1	D2	D3	D4	D5
0	2	5/10	-	-	-	-
1	10	5/10	5/10	5/10	5/10	5/10
5	10	5/10	5/10	5/10	5/10	5/10
10	10	5/10	5/10	5/10	5/10	5/10

Note: Five fish were randomly collected from two tanks of each treatment (1, 5 and 10 mg Cu/L) at the intervals of 24 h (D1 group), 48 h (D2 group), 72 h (D3 group), 96 h (D4 group), and 120 h (D5 group). The sampling from the control group was conducted at once (after 24 h).
